# Sustainable Bacterial Control of Hatching Eggshells Using Essential Oils

**DOI:** 10.3390/antibiotics13111025

**Published:** 2024-10-31

**Authors:** Igor Rafael Ribeiro Vale, Gabriel da Silva Oliveira, Luana Maria de Jesus, Marcio Botelho de Castro, Concepta McManus, Vinícius Machado dos Santos

**Affiliations:** 1 Laboratory of Poultry Science, Federal Institute of Brasília—Campus Planaltina, Brasília 73380-900, Brazil; 2 Faculty of Agronomy and Veterinary Medicine, University of Brasília, Brasília 70910-900, Brazil; gabriels.unb@gmail.com (G.d.S.O.);; 3 Center for Nuclear Energy in Agriculture (CENA), University of São Paulo, São Paulo 13416-000, Brazil

**Keywords:** egg sanitization, essential oils, green antibacterials, hatching eggs, poultry, sanitizers

## Abstract

**Background:** Decontamination of poultry surfaces through appropriate hygiene and sanitation measures can partially mitigate bacterial problems, as this process does not result in the complete elimination of bacteria. Thus, the remaining bacteria can persist and contaminate eggshells. Therefore, regardless of the rigor of the sanitary standards applied on farms, it is suggested that hatching eggs be subjected to the sanitization process. Here, we investigated the effectiveness of essential oil-based antibacterial agents in sanitizing eggs. **Results:** The results indicated that essential oils from *Cinnamomum cassia* (L.) J. Presl. (CCEO), *Syzygium aromaticum* (L.) Merr. & L.M. Perry (SAEO) and *Cymbopogon nardus* (L.) Rendle (CNEO), at specific concentrations, have antibacterial effects in vitro, reducing the load of mesophilic bacteria and enterobacteria in the eggshell by at least 3 and 2 log_10_ CFU/mL, respectively. **Conclusion:** The adoption of CCEO, SAEO and CNEO to reduce the bacterial load on eggshells could be a favorable change to the conventional protocol of egg sanitization with formaldehyde, especially on farms where sanitary standards are insufficient.

## 1. Introduction

The surface of eggshells can be contaminated by bacteria such as *Escherichia coli* and *Staphylococcus* spp., which may account for 84% and 77% of the present Gram-negative and Gram-positive bacteria, respectively [1]. In addition to antibiotic resistance [1], *Escherichia coli* and *Staphylococcus* spp. (e.g., *Staphylococcus aureus*) may also be associated with the death of chicken embryos [2,3]. Eggs appear to be quite vulnerable to several threats, even biofilm contamination [4]. Regardless of the level of bacterial contamination present on the eggshell, which can be influenced by the location of egg deposition, synthetic chemical sanitation remains the main approach for decontamination. On a global scale, formaldehyde is the predominant active agent [5,6]. Formaldehyde demonstrates cutting-edge effectiveness, surpassing many sanitizers available on the market. However, its efficiency is concomitant with significant toxicity to humans and animals, which raises a red alert in the scientific community [7,8,9]. Advanced antibacterials that combine high efficiency with minimal toxicity represent a strategic attempt to demonstrate to the industry the need to overcome the problems associated with the use of formaldehyde. Hydrogen peroxide and Virkon S are among the potential alternative synthetic chemicals for sanitizing hatching eggs [6]. The viability of a sanitizer must be assessed not only by its efficiency and cost but also, above all, by its impact on human safety. The instruments provided by nature, when used properly, proved to be a preferable way to overcome this unhealthiness. 

Unsurprisingly, essential oils are natural bioactive substances extracted from plants. However, the surprisingly versatile spectrum of their biological potential for both human and animal health [10,11,12] continues to dazzle research centers and industry in general. Although essential oils are considered safe and are linked to benefits for living and non-living organisms, they are not free from toxicity, mainly due to their composition [13,14]. The essential oil extracted from *Cinnamomum cassia* (L.) J. Presl. (cinnamon) plants can be characterized by a clear liquid appearance and a yellow color. It has a characteristic odor and can have a density of 1.053 g/cm³ at 20 °C and a refractive index of 1.609 at 20 °C. This oil is composed of a variety of substances, including cinnamic aldehyde, o-methoxy cinnamaldehyde, cinnamyl acetate and benzaldehyde [15]. The essential oil obtained from the *Syzygium aromaticum* (L.) Merr. & L.M. Perry, syn. *Eugenia caryophyllus* (Spreng.) Bullock & S.G. Harrison (clove) plant is characterized by its clear appearance and color, which varies from pale yellow to yellow. Its density can reach 1.038 g/cm³ at 20 °C, and its refractive index can reach 1.530 at 20 °C. This oil is composed of a chemical configuration that includes 3-allyl-6-methoxyphenol, eugenol acetate, 2-pentanone, 4-hydroxy-4-methyl and caryophyllene [16]. The essential oil extracted from the *Cymbopogon nardus* (L.) Rendle (citronella) plant, like the previous oils, normally has a yellowish color. Its density at 20 °C can reach 0.892 g/cm³, and the refractive index at 20 °C can reach 1.469. The chemical contents of this oil include ammonium carbamate, carbinol, neophytadiene, trans-geraniol, phenol-methoxy, norolean, benzofuran, guaiacol, hexadecen-phytol, β-citronellol, trans-caryophyllene, alphahumulene and valerol [17]. The in vitro antibacterial activities of the three essential oils have been documented in previous studies (Table 1).

The use of essential oils to combat the formation of biofilms by foodborne pathogenic bacteria has shown great promise [26]. In this context, studies have also demonstrated that *Cinnamomum cassia* (CCEO), *Syzygium aromaticum* (SAEO) and *Cymbopogon nardus* (CNEO), applied directly or incorporated as active ingredients in biopolymeric films and coatings before application to food, can ensure microbiological quality and food preservation [27,28,29]. These three essential oils have been classified as Generally Recognized as Safe (GRAS) [30,31]. The present study aims to elucidate the antibacterial profiles of CCEO, SAEO and CNEO when they are applied to the surface of eggshells. Previous studies have demonstrated the in vitro antibacterial benefits of these essential oils. Our objective is to expand this knowledge by investigating its antibacterial properties in conditions closer to practical applications for sanitizing hatching eggs. 

## 2. Materials and Methods

CCEO, SAEO and CNEO were obtained commercially (BioEssência®, São Paulo, Brazil). The specifications for each essential oil are described in Table 2. After acquisition, the essential oils, stored in appropriate vials, were maintained in a controlled refrigeration chamber at 4 °C until use.

In summary, sterile white discs of 6 mm impregnated with 10 µL of CCEO, SAEO, or CNEO, both pure and diluted in dimethyl sulfoxide (600–0.005 mg/mL), were evaluated for their ability to inhibit the growth of *Escherichia coli* (ATCC 25922; *E. coli*) and *Staphylococcus aureus* (ATCC 25923; *S. aureus*) via the disc diffusion method [32]. First, the bacteria were activated in brain heart infusion broth and incubated for 24 h at 36 °C. After this period, each bacterial inoculum was standardized in a sterile saline solution (0.85% NaCl) to a turbidity equivalent to 0.5 on the McFarland scale. Then, we tested whether the essential oils, diluted in dimethyl sulfoxide at a concentration of 600 mg/mL, exhibited activity against both strains, using this as the starting point for further dilutions. After confirming activity, serial dilutions were performed to the greatest extent possible. For control purposes, discs impregnated with 15 µg of azithromycin (Laborclin, Paraná, Brazil) and discs impregnated with pure dimethyl sulfoxide were also used. The inhibitory efficacy of each concentration was tested in triplicate and determined by the presence of inhibition zones after 24 h of incubation of the plates at 36 °C.

The inhibitory concentrations of the essential oils, which were determined via the disk diffusion method, were subjected to a broth dilution technique [33] to confirm their efficacy, which was carried out in triplicate. These serial dilutions of essential oils were prepared in test tubes with Mueller–Hinton broth and accompanied by a negative control, which was free of any essential oil. The tubes were incubated at 36 °C for a period of 24 h. After incubation, bacterial growth inhibition was assessed based on the presence or absence of turbidity [33].

The eggs used in this study were non-fertile, an intentional choice considering that the focus was exclusively on external microbiological parameters. The use of non-fertile eggs eliminates any variables associated with embryonic development and, therefore, does not require approval from animal ethics committees. However, this approach allows us to gain clear insight into the relationship between the effects of essential oils on the bacteria present in the shell, with potential implications for embryo protection in future studies. These eggs were collected and selected from a poultry production system of the Embrapa 051 lineage (Planaltina, Federal District, Brazil). The eggs were randomly distributed into six different treatment groups, as described in Table 3. 

Sanitizers based on essential oils were formulated by diluting the oils in 93.8% grain alcohol, using the lowest concentration that demonstrated efficacy against both bacteria tested in the in vitro assay (see results topic). As this alcohol acted as a vehicle for essential oils, its isolated use was also tested in eggs. The sanitizers were individually sprayed on the eggs (~2.5–3 mL/egg) until the entire shell was completely covered.

For an accurate assessment of total aerobic mesophilic bacteria and Enterobacteriaceae, six eggs from a non-sanitized group and sanitized groups were subjected to a washing procedure. Each egg was washed individually in sterile plastic bags (Labplas, Quebec, Canada) containing 75 mL of 0.1% peptone water for a period of 2 minutes. With the egg submerged in water and the bag sealed, the egg was gently massaged over its entire surface. The solutions resulting from washing were then subjected to serial decimal dilutions in 0.1% peptone water solution. Subsequently, aliquots of 100 µL of each dilution were seeded via the Drigalsky loop spreading technique in sextuplicate on plates containing count agar (Laborclin, Paraná, Brazil) and violet red bile glucose agar (Laborclin, Paraná, Brazil). The plates were incubated at 36 °C for 48 h. The colonies were counted and the results were log_10_ transformed.

For in vitro antibacterial analysis and bacterial counts in eggshells, a completely randomized design was used with triplicate and sextuplicate samples, respectively. The results of the microbiological analyses were subjected to an analysis of variance (PROC GLM) and compared via the Tukey test, with a significance level of 5%. The entire statistical process was conducted in SAS Studio 9.4 University Edition software (SAS Institute Inc., Cary, NC, USA).

## 3. Results

This study investigated the inhibition potential of three essential oils against *E. coli* and *S. aureus*. The concentrations of CCEO, SAEO and CNEO, which are capable of inhibiting both bacterial strains, were determined via the disk diffusion method and were between 600 and 0.59 mg/mL, 600 and 4.69 mg/mL and 600 and 0.59 mg/mL, respectively (Table 4). The average inhibition zone for the positive control, azithromycin (15 µg), was 23.73 ± 2.43 mm for *E. coli* and 21.00 ± 1.73 mm for *S. aureus*. To ensure the reliability of the results obtained via the disk diffusion method, the concentrations of each essential oil that inhibited both bacteria were also subjected to the broth dilution method. The results demonstrated a complete absence of bacterial growth at all concentrations tested, except in the negative controls (without the presence of diluted essential oil). The minimum inhibitory concentrations of CCEO, SAEO and CNEO, which were determined to be 0.59 mg/mL, 4.69 mg/mL and 0.59 mg/mL, respectively, were selected to evaluate their effectiveness in the sanitization of eggshells.

The eggshells analyzed presented counts of aerobic mesophilic bacteria ranging from 4.96 to 1.01 log_10_ CFU/mL (Table 5). Sanitizing eggs with essential oils reduced eggshell bacterial levels by at least 3 log_10_ of non-sanitized eggs. The enterobacterial count ranged from 2.24 to 1.20 log_10_ CFU/mL. Eggs treated with essential oils did not show a detectable presence of enterobacteria, indicating superior efficacy compared with formaldehyde in eliminating this bacterial group.

## 4. Discussion

The antibacterial activity of essential oils is of scientific interest, as proven by the substantial number of published studies. A recent analysis in the Scopus database, which uses the terms "(TITLE-ABS-KEY (antibacterial AND activity AND essential AND oils) OR TITLE-ABS-KEY (poultry)", revealed a total of 60,526 publications in the last five years. When the search was restricted with the keyword "poultry and eggs", we identified 17,180 publications. These data highlight the appreciation of the antibacterial benefits of essential oils in poultry. This trend highlights the commitment of research centers to officially integrate these natural compounds into poultry production. One of the objectives pursued by researchers is to encourage the use of essential oils in the sanitization of hatching eggs [35].

In this study, essential oils inhibited both Gram-positive and Gram-negative bacterial strains. Here, we recorded significant inhibition halos against *E. coli* and *S. aureus* on discs impregnated with CCEO, SAEO and CNEO via the disc diffusion method. These results were corroborated by the broth dilution method. These results are in line with previous studies that, in addition to CCEO, SAEO and CNEO, also documented the in vitro antibacterial effectiveness of other essential oils against the same bacterial strains [18,19,22,24,25,36]. Aouadhi et al. [37] suggested that essential oils can induce significant disturbances in the permeability and integrity of the bacterial cell membrane. These effects result in the leakage of DNA and proteins from the bacterial cytoplasm. Many existing publications reveal that Gram-negative bacteria are perceived as more resistant to essential oils because of their morphological structure, particularly the presence of an external lipopolysaccharide membrane [38]. However, the results obtained in this study challenge this conception, indicating a greater sensitivity of Gram-negative bacteria than of Gram-positive bacteria when exposed to essential oils. This finding reveals the need to avoid hasty generalizations when one bacterial group is more sensitive to essential oils than another. For example, while Lopez-Romero et al. [39] observed that Gram-positive bacteria were more resistant to essential oils, Ribeiro et al. [40] found that Gram-negative bacteria were more resistant.

CCEO, SAEO and CNEO have demonstrated efficacy in inhibiting bacterial growth on eggshells. In the study by Oliveira et al. [41] on the bacteriological count of eggs sanitized with essential oils from *Ocimum basilicum*, *Citrus aurantifolia* and *Allium sativum*, the counts of mesophilic bacteria ranged from 3.09 ± 0.23 to 1.87 ± 0.54 log_10_ CFU/mL. No enterobacteria were detected in eggs sanitized with *Citrus aurantifolia* or *Allium sativum*, whereas for *Ocimum basilicum*, the enterobacterial count was 1.02 ± 0.89 log_10_ CFU/mL. All the results obtained for eggs sanitized with essential oils were significantly lower than those obtained for non-sanitized eggs, which presented mesophilic bacteria counts of 5.12 ± 0.10 log_10_ CFU/mL and enterobacteria counts of 3.25 ± 0.75 log_10_ CFU/mL. The effectiveness of essential oils in reducing bacteriological counts in eggshells was also reported in the study by Mustafa et al. [42]. Bacteria can become nonviable when they interact with essential oils, as previously stated by Aouadhi et al. [37].

Our sustainable plan for sanitizing eggs using essential oils, similar to industry practices, provides cutting-edge training and paves the way for future commercial applications. This plan proposes a viable and conscientious solution based on renewable resources for natural sanitization in poultry farming. It addresses the demand for solutions that minimize environmental impacts while also providing opportunities to promote and encourage the development of a more sustainable poultry sanitizer market. In addition to its proven effectiveness in reducing bacterial load on eggshells, several factors may favor the adoption of this natural approach to the sanitization of hatching eggs. The first factor is the commercial availability of essential oils [43]. The second is the potential to establish partnerships between poultry producers and aromatic plant growers, fostering the cultivation and sourcing of essential raw materials to produce these sanitizers. Although essential oils may have a higher cost compared to some synthetic products, their high efficiency at low concentrations can mitigate costs that might otherwise hinder their use [44]. The small quantity required to achieve the expected antibacterial efficacy in the process enhances their economic attractiveness.

## 5. Conclusions

The CCEO, SAEO and CNEO showed potential as sanitizing agents, providing an option to ensure that eggshell surfaces have a lower bacterial load, possibly including potentially pathogenic bacteria. In reducing mesophilic bacteria, CCEO was 73.39% more effective than non-sanitized eggs and 34.98% more effective than formaldehyde; SAEO was 65.73% more effective than non-sanitized eggs and 16.26% more effective than formaldehyde; CNEO was 79.64% more effective than non-sanitized eggs and 50.25% more effective than formaldehyde. This proposal aims to overcome the disadvantages associated with conventional sanitization methods that use formaldehyde/paraformaldehyde. Although CCEO, SAEO and CNEO have demonstrated antibacterial efficacy, there are still outstanding questions that require in-depth investigation, especially concerning their safety and economic viability. As essential oils are multifunctional compounds and the sanitizers that have them as active ingredients are simple to prepare and apply, and considering that some essential oil-based sanitizers have already demonstrated potential benefits, the widespread adoption of their use in hatching egg sanitization protocols would reduce the need on unsustainable products and effectively address contamination issues in poultry farming. Furthermore, the use of these natural compounds could simultaneously integrate the demands of other stages of poultry production, as well as the industrial sectors responsible for manufacturing these sanitizers, benefiting both small and large producers. In addition to natural sanitization tests at the laboratory level, researchers are encouraged to carry out future studies that evaluate its effectiveness on an industrial scale.

## Figures and Tables

**Table 1:** Summary of the in vitro antibacterial activities of *Cinnamomum cassia* (CCEO), *Syzygium aromaticum* (SAEO) and *Cymbopogon nardus* (CNEO) essential oils.

Essential Oil	Concentration/Dose	Sensitive Bacteria	Study
CCEO	100 μL	*Pseudomonas aeruginosa*	[18]
		*Staphylococus aureus*	
		*Escherichia coli*	
		*Klebsiella pneumoniae*	
	10 µL	*Staphylococus aureus*	[19]
		*Salmonella typhimurium*	
	1.13 mg/mL	*Streptococcus mutans*	[20]
	0.56 mg/mL	*Staphylococcus aureus*	
SAEO	10%	*Staphylococcus aureus*	[21]
		*Escherichia coli*	
		*Salmonella abony*	
	40 µL	*Staphylococus aureus*	[22]
		*Escherichia coli*	
		*Klebsiella pneumoniae*	
		*Pseudomonas aeruginosa*	
CNEO	31.25 µg/mL	*Staphylococcus aureus*	[23]
		*Listeria monocytogenes*	
	250 µg/mL	*Pseudomonas aeruginosa*	
	125 µg/mL	*Salmonella choleraesuis*	
	0.06%	*Staphylococcus aureus*	[24]
	2.5%	*Pseudomonas aeruginosa*	
	3.13%	*Escherichia coli*	[25]
	6.25%	*Pseudomonas aeruginosa*	

**Table 2:** Specifications of commercially acquired essential oils from *Cinnamomum cassia* (CCEO), *Syzygium aromaticum* (SAEO) and *Cymbopogon nardus* (CNEO).

Essential Oil	Extraction Method	Density (20 °C)	Refraction Index (20 °C)	Main Chemical Compound
CCEO	Steam distillation of leaves, stems and peel	1.044	1.604	Cinnamaldehyde—80.15%
SAEO	Steam distillation of the leaves	1.044	1.533	Eugenol—83.62%
CNEO	Steam distillation of the leaves	0.880	1.466	Citronellal—43.34%

**Table 3:** Details of egg application procedures.

Sanitizers	Sanitizer Concentration	Manual Application Method	Number of Eggs	Egg Drying Period
NE			6	30 min
GA	93.8%	Spraying	6	30 min
FA	1.5% *	Spraying	6	30 min
CCEO	0.59 mg/mL	Spraying	6	30 min
SAEO	4.69 mg/mL	Spraying	6	30 min
CNEO	0.59 mg/mL	Spraying	6	30 min

* FA, Formaldehyde (36.5–38% content) was diluted in distilled water, reaching a concentration of 1.5%, as described by Al-Shemery and Kamaluddin [34]. The essential oils were diluted in grain alcohol. NE, non-sanitized eggs; GA, grain alcohol; CCEO, *Cinnamomum cassia* essential oil; SAEO, *Syzygium aromaticum* essential oil; CNEO, *Cymbopogon nardus* essential oil.

**Table 4:** Inhibition halos of essential oils against the two bacterial strains.

Concentrations (mg/mL)	*E. coli*			*S. aureus*		
	CCEO	SAEO	CNEO	CCEO	SAEO	CNEO
	**Inhibition Halos (mm)**					
600	33.33 ± 3.05	15.33 ± 1.53	34.67 ± 3.05	30.33 ± 1.53	11.33 ± 1.53	32.00 ± 5.29
300	30.00 ± 1.00	14.33 ± 0.58	31.33 ± 6.11	30.00 ± 1.00	10.67 ± 0.58	31.33 ± 1.15
150	29.33 ± 3.21	14.33 ± 0.58	31.00 ± 1.15	29.00 ± 0.00	10.33 ± 1.15	23.33 ± 3.05
75	29.00 ± 1.73	11.67 ± 0.58	25.33 ± 4.73	26.33 ± 0.58	8.67 ± 0.58	20.00 ± 2.00
37.5	27.00 ± 3.61	10.00 ± 0.00	21.33 ± 1.15	25.33 ± 2.31	8.67 ± 2.08	20.00 ± 4.00
18.75	23.00 ± 4.00	9.00 ± 2.00	20.67 ± 1.15	18.67 ± 2.31	8.33 ± 2.31	19.33 ± 4.16
9.38	16.33 ± 5.13	8.67 ± 1.53	19.33 ± 1.15	17.00 ± 3.46	8.00 ± 1.00	19.33 ± 4.62
4.69	13.67 ± 3.06	7.67 ± 1.15	17.33 ± 2.31	10.00 ± 2.00	7.67 ± 1.15	18.00 ± 2.00
2.34	11.00 ± 1.73	7.67 ± 1.53	16.33 ± 4.73	8.67 ± 3.79	ND	18.67 ± 1.15
1.17	10.67 ± 2.08	ND	14.67 ± 2.08	8.33 ± 2.08	ND	10.67 ± 0.58
0.59	9.67 ± 1.53	ND	10.33 ± 1.15	8.00 ± 1.73	ND	9.67 ± 2.52
0.29	9.00 ± 2.83	ND	ND	ND	ND	9.33 ± 0.58
0.15	8.67 ± 1.15	ND	ND	ND	ND	ND
0.07	8.33 ± 0.58	ND	ND	ND	ND	ND
0.037	7.33 ± 0.58	ND	ND	ND	ND	ND
0.018	ND	ND	ND	ND	ND	ND
0.009	ND	ND	ND	ND	ND	ND
0.005	ND	ND	ND	ND	ND	ND

Abbreviations: CCEO, *Cinnamon cassia* essential oil; SAEO, *Syzygium aromaticum* essential oil; CNEO, *Cymbopogon nardus* essential oil; ND, No inhibition halo detected.

**Table 5:** Bacterial counts on eggshells after sanitization protocols ^1^.

Treatments	Total Aerobic Mesophilic Bacteria (log_10_ CFU/mL)	Enterobacteriaceae (log_10_ CFU/mL)
NE	4.96 ± 0.52 ^a^	2.24 ± 1.20 ^a^
GA	4.06 ± 0.50 ^a^	2.02 ± 0.41 ^a^
FA	2.03 ± 0.47 ^b^	1.20 ± 1.31 ^ab^
CCEO	1.32 ± 0.85 ^b^	0.00 ± 0.00 * ^b^
SAEO	1.70 ± 0.83 ^b^	0.00 ± 0.00 ^b^
CNEO	1.01 ± 0.70 ^b^	0.00 ± 0.00 ^b^
*p* value	<0.0001	<0.0001

Abbreviations: NE, non-sanitized eggs; GA, grain alcohol; FA, formaldehyde; CCEO, *Cinnamomum cassia* essential oil; SAEO, *Syzygium aromaticum* essential oil; CNEO, *Cymbopogon nardus* essential oil. ^1^ Data are expressed as the mean (log_10_ CFU/mL) ± standard deviation. * Indicates a count below the detection limit of <10 CFU/mL. Different letters in the same column indicate significant differences among means (*p* < 0.05).

## Data Availability

The raw data supporting the conclusions of this article will be made available by the authors on request.

## References

[1] Verma S., Wadhwa N.K., Bajaj D., Sharma R., Rajni E., Priyadarshani A. (2023). A Microbiological Analysis of Egg Shell Bacteria. Vantage J. Themat. Anal..

[2] Kabir S.M.K. (2010). Avian Colibacillosis and Salmonellosis: A Closer Look at Epidemiology, Pathogenesis, Diagnosis, Control and Public Health Concerns. Int. J. Environ. Res. Public Health.

[3] Kosecka-Strojek M., Trzeciak J., Homa J., Trzeciak K., Władyka B., Trela M., Międzobrodzki J., Lis M.W. (2021). Effect of *Staphylococcus aureus* Infection on The Heat Stress Protein 70 (HSP70) Level in Chicken Embryo Tissues. Poult. Sci..

[4] Almeida e Silva T., Gorup L.F., de Araújo R.P., Fonseca G.G., Martelli S.M., de Oliveira K.M.P., Faraoni L.H., de Arruda E.G.R., Gomes R.A.B., da Silva C.H.M. (2020). Synergy of Biodegradable Polymer Coatings With Quaternary Ammonium Salts Mediating Barrier Function against Bacterial Contamination and Dehydration of Eggs. Food Bioprocess Technol..

[5] Melo E.F., Clímaco W.L.S., Triginelli M.V., Vaz D.P., De Souza M.R., Baião N.C., Pompeu M.A., Lara L.J.C. (2019). An evaluation of alternative methods for sanitizing hatching eggs. Poult. Sci..

[6] Oliveira G.D.S., McManus C., Salgado C.B., Dos Santos V.M. (2022). Effects of sanitizers on microbiological control of hatching eggshells and poultry health during embryogenesis and early stages after hatching in the last decade. Animals.

[7] Cadirci S. (2009). Disinfection of Hatching Eggs by Formaldehyde Fumigation-a Review. Eur. Poult. Sci..

[8] Hayretdag S., Kolankaya D. (2008). Investigation of the Effects of Preincubation Formaldehyde Fumigation on the Tracheal Epithelium of Chicken Embryos and Chicks. Turk. J. Vet. Anim. Sci..

[9] Zeweil H.S., Rizk R.E., Bekhet G.M., Ahmed M.R. (2015). Comparing the Effectiveness of Egg Disinfectants against Bacteria and Mitotic Indices of Developing Chick Embryos. J. Basic Appl. Zool..

[10] Ezzat Abd El-Hack M., Alagawany M., Ragab Farag M., Tiwari R., Karthik K., Dhama K., Zorriehzahra J., Adel M. (2016). Beneficial Impacts of Thymol Essential Oil on Health and Production of Animals, Fish and Poultry: A Review. J. Essent. Oil Res..

[11] Chen Y., Liu L., Wang H., Ma J., Peng W., Li X., Lai Y., Zhang B., Zhang D. (2021). Environmentally Friendly Plant Essential Oil: Liquid Gold for Human Health. Adv. Agron..

[12] Oliveira G.D.S., McManus C., de Araújo M.V., de Sousa D.E.R., de Macêdo I.L., Castro M.B.D., Santos V.M.D. (2023). Sanitizing hatching eggs with essential oils: Avian and microbiological safety. Microorganisms.

[13] Wojtunik-Kulesza K.A. (2022). Toxicity of Selected Monoterpenes and Essential Oils Rich in These Compounds. Molecules.

[14] Vostinaru O., Heghes S.C., Filip L. (2020). Safety Profile of Essential Oils. Essential Oils-Bioactive Compounds, New Perspectives and Applications.

[15] Bongiovanni V., Colombo M., Laura C., Andrea T., Daniela C. (2017). Determining Odour-Active Compounds in a Commercial Sample of *Cinnamomun cassia* Essential Oil Using GC–MS and GC-O. J. Chromatogr. Sep. Tech..

[16] Uddin M.A., Shahinuzzaman M., Rana M.S., Yaakob Z. (2017). Study of chemical composition and medicinal properties of volatile oil from clove buds (*Eugenia caryophyllus*). Int. J. Pharm. Sci. Res..

[17] Saputra N.A., Wibisono H.S., Darmawan S., Pari G. (2020). Chemical Composition of *Cymbopogon Nardus* Essential Oil and Its Broad-*Spectrum* Benefit. IOP Conference Series: Earth and Environmental Science.

[18] Moreira Silva A.R., Lopes Mendes L.D.S., Silva de Souza E.F., Luz Pereira M., Silva Alves M., Pará Alves E.V., Lima Torres E., Gaspar Novais T.M. (2023). Avaliação da Atividade Antimicrobiana do Óleo Essencial de *Cinnamomum cassia*. Rev. Foco (Interdiscip. Stud. J.).

[19] Penteado A.L., Eschionato R.A., de Souza D.R.C., Queiroz S.D.N. (2021). Avaliação in vitro de atividade antimicrobiana de óleos essenciais contra *Salmonella typhimurium* e *Staphylococcus aureus*. Hig. Aliment..

[20] Freire I.C.M., Pérez A.L.A.L., Cardoso A.M.R., Mariz B.A.L.A., Almeida L.F.D., Cavalcanti Y.W., Padilha W.W.N. (2014). Atividade antibacteriana de Óleos Essenciais sobre *Streptococcus mutans* e *Staphylococcus aureus*. Rev. Bras. Plant. Med..

[21] Itaparica N.V.D.A. Avaliação da Atividade Antimicrobiana dos Óleos Essenciais de Eugenia caryophyllata e Croton rhamnifolioides pax e hoffm. Proceedings of the VII CONNEPI-Congresso Norte Nordeste de Pesquisa e Inovação.

[22] Oliveira A.F.M., da Silva F.L., Morais F.M., da Silva R.T., dos Santos R.R.L., da Silva L.L.W.V., Oliveira J.M., Morais C.C., Cesar K.K.F.A. (2022). Atividade Antimicrobiana de Óleos Essenciais Frente a Bactérias Patogênicas de Importância Clínica. Res. Soc. Dev..

[23] Andrade M.A., dasGraças Cardoso M., Batista L.R., Mallet A.C., Machado S.M. (2012). Essential Oils of *Cinnamomum zeylanicum*, *Cymbopogon nardus* and *Zingiber officinale*: Composition, Antioxidant and Antibacterial Activities. Rev. Ciên. Agron..

[24] Santos C.H.D.S., Piccoli R.H., Tebaldi V.M.R. (2017). Atividade Antimicrobiana de Óleos Essenciais e Compostos Isolados Frente aos Agentes Patogênicos de Origem Clínica e Alimentar. R. Inst. Adolfo Lutz.

[25] Contrucci B.A., Silva R., Junior R.A., Kozusny-Andreani D.I. (2019). Efeito de Óleos Essenciais Sobre Bactérias Gram-Negativas Isoladas de Alimentos. Ens. Ciên. Ciên. Biol. Agr. Saúde..

[26] Kolypetri S., Kostoglou D., Nikolaou A., Kourkoutas Y., Giaouris E. (2023). Chemical Composition, Antibacterial and Antibiofilm Actions of Oregano (*Origanum vulgare* subsp. Hirtum) Essential Oil against *Salmonella typhimurium* and *Listeria monocytogenes*. Foods.

[27] Ferreira R.R., Souza A.G., Quispe Y.M., Rosa D.S. (2021). Essential Oils Loaded-Chitosan Nanocapsules Incorporation in Biodegradable Starch Films: A Strategy to Improve Fruits Shelf Life. Int. J. Biol. Macromol..

[28] Motelica L., Ficai D., Oprea O., Ficai A., Trusca R.-D., Andronescu E., Holban A. (2021). Biodegradable Alginate Films with ZnO Nanoparticles and Citronella Essential Oil—A Novel Antimicrobial Structure. Pharmaceutics.

[29] Kačániová M., Garzoli S., Ben Hsouna A., Ban Z., Elizondo-Luevano J.H., Kluz M.I., Ben Saad R., Haščík P., Čmiková N., Waskiewicz-Robak B. (2024). Enhancing Deer Sous Vide Meat Shelf Life and Safety with *Eugenia caryophyllus* Essential Oil against *Salmonella enterica*. Foods.

[30] Food and Drug Administration, Department of Health and Human Services Subchapter B—Food for Human Consumption (Continued). Part 182—Substances Generally Recognized as Safe. Subpart A—General Provisions. Sec. 182.20 Essential Oils, Oleoresins (Solvent-Free), and Natural Extractives (Including Distillates). https://www.accessdata.fda.gov/scripts/cdrh/cfdocs/cfcfr/cfrsearch.cfm?fr=182.20..

[31] Food and Drug Administration, Department of Health and Human Services Subchapter B—Food for Human Consumption (Continued). PART 184—Direct Food Substances Affirmed as Generally Recognized as Safe. Subpart B—Listing of Specific Substances Affirmed as GRAS. Sec. 184.1257 Clove and Its Derivatives. https://www.accessdata.fda.gov/scripts/cdrh/cfdocs/cfcfr/CFRSearch.cfm?fr=184.1257.

[32] Bauer A.W., Kirby W.M.M., Sherris J.C., Turck M. (1966). Antibiotic Susceptibility Testing by a Standardized Single Disk Method. Am. J. Clin. Pathol..

[33] Wiegand I., Hilpert K., Hancock R.E. (2008). Agar and Broth Dilution Methods to Determine the Minimal Inhibitory Concentration (MIC) of Antimicrobial Substances. Nat. Protoc..

[34] Al-Shemery N.J., Kamaluddin Z.N. (2018). Effect of Using Different Concentrations of Hydrogen Peroxide and Formalin Compared to Formaldehyde Evaporation in Sterilization of Hatching Eggs of Broiler. Euphrates J. Agric. Sci..

[35] Oliveira G.D.S., dos Santos V.M., Nascimento S.T. (2021). Essential Oils as Sanitisers for Hatching Eggs. Worlds Poult. Sci. J..

[36] Puvača N., Milenković J., Galonja Coghill T., Bursić V., Petrović A., Tanasković S., Pelić M., Ljubojević Pelić D., Miljković T. (2021). Antimicrobial Activity of Selected Essential Oils against Selected Pathogenic Bacteria: In Vitro Study. Antibiotics.

[37] Aouadhi C., Jouini A., Maaroufi K., Maaroufi A. (2024). Antibacterial Effect of Eight Essential Oils against Bacteria Implicated in Bovine Mastitis and Characterization of Primary Action Mode of *Thymus capitatus* Essential Oil. Antibiotics.

[38] Fournomiti M., Kimbaris A., Mantzourani I., Plessas S., Theodoridou I., Papaemmanouil V., Kapsiotis I., Panopoulou M., Stavropoulou E., Bezirtzoglou E.E. (2015). Antimicrobial Activity of Essential Oils of Cultivated Oregano (*Origanum vulgare*), Sage (*Salvia officinalis*), and Thyme (*Thymus vulgaris*) against Clinical Isolates of *Escherichia coli*, *Klebsiella oxytoca*, and *Klebsiella pneumoniae*. Microb. Ecol. Health Dis..

[39] Lopez-Romero J.C., González-Ríos H., Borges A., Simõs M. (2015). Antibacterial Effects and Mode of Action of Selected Essential Oils Components against *Escherichia coli* and *Staphylococcus aureus*. Evid. Based Complementary Altern. Med..

[40] Ribeiro O.S., Fontaine V., Mathieu V., Zhiri A., Baudoux D., Stévigny C., Souard F. (2020). Antibacterial and cytotoxic activities of ten commercially available essential Oils. Antibiotics.

[41] Oliveira G.D.S., McManus C., Vale I.R.R., Dos Santos V.M. (2024). Obtaining Microbiologically Safe Hatching Eggs from Hatcheries: Using Essential Oils for Integrated Sanitization Strategies in Hatching Eggs, Poultry Houses and Poultry. Pathogens.

[42] Mustafa A.A., Mirza R.A., Aziz H.I. (2023). Lavender Essential Oil in Sanitation on Fertile Egg. Passer J. Basic Appl. Sci..

[43] Bizzo H.R., Rezende C.M. (2022). O mercado de óleos essenciais no Brasil e no mundo na última década. Quím. Nova..

[44] Chouhan S., Sharma K., Guleria S. (2017). Antimicrobial Activity of Some Essential Oils-Present Status and Future Perspectives. Medicines.

